# Supplementary material for: Humanized NSG Mouse Models as a Preclinical Tool for Translational Research in Inflammatory Bowel Diseases
Source: Int J Mol Sci. 2023 Aug 2;24(15):12348. doi: 10.3390/ijms241512348 (PMC10418464; doi:10.3390/ijms241512348)
Supplement: Supplementary file 1 [file ijms-24-12348-s001.zip › Table S2. Antibodies used for IHC ICC.pdf]

Table S1. Antibodies used for immune-histochemistry and immune -cytochemistry

**Surfacemarker**	**Colour**	**Cat # RRID #**
Anti-hu CD4 RPA-T4		ThemoFisher Scientific Cat#14-0049-82, RRID:AB_467077
Anti-hu CD8		Scientific Cat# 14-0008-82, RRID:AB_2572848
Anti-hu CD14		Thermo Fisher Scientific Cat# 14-0149-82, RRID:AB_467129
Anti-hu CD19		Thermo Fisher Scientific Cat# 14-0190-82, RRID:AB_11219274
Anti hu CD45		BioLegend Cat# 304002, RRID:AB_314390)
Anti TRPA1		Alomone Labs Cat# ACC-037, RRID:AB_2040232
Anti ms Vimentin		Thermo Fisher Scientific Cat# MA5-11883, RRID:AB_10985392
Anti Col1A1		Thermo Fisher Scientific Cat# PA5-29569, RRID:AB_2547045
Anti αSMA		Thermo Fisher Scientific Cat# 14-9760-82, RRID:AB_2572996
Mouse IgG1 Isotype control		Thermofisher Thermo Fisher Scientific Cat# 14-4714-82, RRID:AB_470111
Rabbit Isotype control		Thermo Fisher Scientific Cat# A-11059, RRID:AB_2534106
Goat anti rabbit	Alexa Fluor 647	Thermo Fisher Scientific Cat# A-21244, RRID:AB_2535812
Rabbit anti mouse	Alexa Fluor 488	Thermo Fisher Scientific Cat# A-11059, RRID:AB_2534106
